# High Homocysteine-Thiolactone Leads to Reduced MENIN Protein Expression and an Impaired DNA Damage Response: Implications for Neural Tube Defects

**DOI:** 10.1007/s12035-024-04033-7

**Published:** 2024-02-22

**Authors:** Baoling Bai, Chunlei Wan, Zonghui Xiao, Dan Li, Lingyun Liu, Kexin Zhang, Ting Zhang, Qin Zhang

**Affiliations:** 1 Beijing Municipal Key Laboratory of Child Development and Nutriomics, Capital Institute of Pediatrics, Yabao Road 2, Beijing, 100020 China; 2 Department of Pediatrics, Tongzhou Maternal and Child Health Care Hospital, Beijing, 101101 China

**Keywords:** Neural tube defects, Hyperhomocysteinemia (HHcy), Homocysteine-thiolactone (HTL), *Men1*/Menin, Histone 3 lysine 4 trimethylation (H3K4me3), DNA damage response (DDR), Histone 3 lysine 79 homocysteinylation (H3K79hcy)

## Abstract

DNA damage is associated with hyperhomocysteinemia (HHcy) and neural tube defects (NTDs). Additionally, HHcy is a risk factor for NTDs. Therefore, this study examined whether DNA damage is involved in HHcy-induced NTDs and investigated the underlying pathological mechanisms involved. Embryonic day 9 (E9) mouse neuroectoderm cells (NE4C) and homocysteine-thiolactone (HTL, active metabolite of Hcy)-induced NTD chicken embryos were studied by Western blotting, immunofluorescence. RNA interference or gene overexpression techniques were employed to investigate the impact of Menin expression changes on the DNA damage. Chromatin immunoprecipitation-quantitative polymerase chain reaction was used to investigate the epigenetic regulation of histone modifications. An increase in γH2AX (a DNA damage indicator) was detected in HTL-induced NTD chicken embryos and HTL-treated NE4C, accompanied by dysregulation of phospho-Atr-Chk1-nucleotide excision repair (NER) pathway. Further investigation, based on previous research, revealed that disruption of NER was subject to the epigenetic regulation of low-expressed Menin-H3K4me3. Overexpression of Menin or supplementation with folic acid in HTL-treated NE4C reversed the adverse effects caused by high HTL. Additionally, by overexpressing the *Mars* gene, we tentatively propose a mechanism whereby HTL regulates Menin expression through H3K79hcy, which subsequently influences H3K4me3 modifications, reflecting an interaction between histone modifications. Finally, in 10 human fetal NTDs with HHcy, we detected a decrease in the expression of Menin-H3K4me3 and disorder in the NER pathway, which to some extent validated our proposed mechanism. The present study demonstrated that the decreased expression of Menin in high HTL downregulated H3K4me3 modifications, further weakening the Atr-Chk1-NER pathway, resulting in the occurrence of NTDs.

**Supplementary Information:**

The online version contains supplementary material available at 10.1007/s12035-024-04033-7.

## Introduction

Elevated plasma concentrations of total homocysteine are defined as hyperhomocysteinemia (HHcy) [1], which has been linked to the induction of neural tube defects (NTDs) [2]. HHcy is caused by a maternal suboptimal intake of the nutrient folate, leading to the dysregulated activities of methionine and folate metabolism [2, 3]. NTDs can be mitigated by reducing total homocysteine levels through folic acid (FA) supplementation [1]. However, the precise mechanisms underlying HHcy-induced NTDs and the protective effects exerted by FA remain poorly understood.

The pathology of NTDs is linked to elevated reactive oxygen species (ROS) levels [4], DNA damage [5, 6], and cell-cycle arrest [7]. These effects parallel those of HHcy, which increases ROS levels [8, 9], promotes DNA damage [10], and induces G1 cell-cycle arrest [11]. Additionally, impaired DNA damage repair and genomic instability are common in NTDs and HHcy [12–14]. To maintain genome integrity under normal conditions, the ataxia-telangiectasia mutated (ATM)-checkpoint kinase 2 (Chk2) DNA damage response (DDR) pathway is activated by DNA double-strand breaks, and the ataxia-telangiectasia and rad3-related protein (ATR)-checkpoint kinase 1 (Chk1) DDR pathway can be activated by nucleotide excision and DNA mismatch [15]. The activated ATR-Chk1 pathway triggers a complex signaling network, which plays a crucial role in checkpoint control during DNA damage, and causes cell cycle pause to allow sufficient time for nucleotide excision repair (NER) [16]. Specifically, this pathway can directly drive NER by phosphorylating and activating xeroderma pigmentosum group A (XPA). Subsequently, the XPA that recognizes the damaged sites interacts with other components of the NER pathway such as excision repair 8 (Ercc8), DNA damage binding protein 1 (Ddb1), cullin 4A (Cul4a), ubiquitin specific peptidase 7 (Usp7), and DNA ligase 1 (Lig1), to facilitate the repair of DNA damage [17–20]. Wang et al. discovered that HHcy impairs ATR, causing cell-cycle checkpoint and DDR dysregulation, thereby suppressing DNA damage repair [14, 21]. Collectively, these findings suggest that HHcy may induce NTDs through the dysregulation of DDR.

A potential mechanism of HHcy toxicity involves the non-enzymatic binding of homocysteine thiolactone (HTL) (a highly reactive hcy metabolite is generated by methionyl-tRNA synthetases (MARS)) to intracellular and extracellular proteins during the post-translational process. This forms protein lysine (N)-homocysteinylation modifications, which impair protein function and disrupt hcy metabolism, thereby exerting adverse effects on cellular function and embryonic development [22–26]. In a FA deficiency-induced NTD mouse model, maternal serum folate levels had a significant negative correlation with serum hcy levels, whereas serum hcy levels had a significant positive correlation with the titer of autoantibodies against homocysteinylated proteins [25], suggesting that a low-folate diet inducing NTDs might increase hcy and protein homocysteinylation. HHcy promotes the N-homocysteinylation of superoxide dismutase-1/2 (SOD1/2) leading to the inactivation of SOD1/2, thereby increasing ROS levels [13]. This provides a new explanation for the oxidative stress induced by HHcy during the development of NTDs. Additionally, histone homocysteinylation has a critical role in the pathogenesis of HHcy-induced NTDs. As demonstrated in our previous study, HTL can elevate H3K79 homocysteinylation levels (H3K79hcy) in a dose-dependent manner in NE4C cells, which further epigenetic regulation of the expression of neural tube closure-related genes such as *Smarca4*, *Cecr2*, and *Dnmt3b* [23]. Our research outcomes have raised a captivating question: Does HTL mediate histone homocysteinylation modifications, thereby triggering an imbalance in DDR? In addition, we also observed that high HTL could potentially impact the methylation of histones [23]. Considering histone H3 lysine 4 trimethylation (H3K4me3) and histone H3 lysine 79 dimethylation (H3K79me2) can epigenetically regulate the transcription of key genes involved in neural tube closure [28, 29], we further propose this question: Does a cooperative effect exist between histone homocysteinylation and methylation modifications in the process of DDR disruption caused by HHcy or high HTL? If so, what is the specific mechanism behind it?

Here, we established HTL-treated NE4C cells and an HTL-induced NTDs chicken embryo model and collected HHcy-related human fetal NTD samples to examine their DNA damage status. By generating *Men1*-overexpressing (*Men1*-OE) NE4C cells, we further explored the epigenetic regulatory mechanisms that mediate genomic stability to elucidate the pathogenesis of high HTL-induced NTDs.

## Methods

### Human Subjects

Ten cases of HHcy-related NTDs and 10 normal control samples were obtained from Lvliang, Shanxi, China. NTDs were diagnosed according to the International Classification of Diseases 10th edition code, with early pregnancy ultrasound screening. The specific phenotypes of NTDs were mostly spina bifida malformations; the main clinical characteristic was incomplete vertebral canal closure in the lower spinal region, resulting in a split spine. Six of which were accompanied by hydrocephalus. Control fetuses matched with HHcy-related NTD group were miscarried due to non-medical reasons (voluntary early abortion for non-health or non-pathological reasons), excluding malformations or fetal intrauterine growth retardation. And the controls and the NTD group were matched in terms of the mother’s age and the gender of the fetus. Both groups of mothers did not take FA supplements (Supplementary Table 1) [23]. We extracted the brain tissue of NTDs and control fetuses for subsequent measurement.

### Hcy Level Detection

Twenty milligrams of fetal brain tissue was treated with 150 mL of 50 mM dithiothreitol for 20 min to reduce disulfide bonds, followed by the addition of 200 µL internal standard (Hcy-d4). After sonication and centrifugation at 12,000 g, the supernatant was transferred to SPE tips. Hcy was dissolved in methanol–water (65:35, v/v) containing 1 mM ammonium formate. An Agilent 6410B mass spectrometer was coupled to an Agilent 1200 system HPLC for sample separation using a Zorbax Bonus-RP column with a flow rate of 0.25 mL/min. The column temperature was set to 35 °C; MS/MS experiments were conducted in ESI + mode, performing MRM monitoring. MRM transitions for Hcy and Hcy-d4 were 350–204.1 and 354.2–208.1, respectively [23].

### HTL Treatment of Chicken Embryos

The samples required for this study originate from chicken embryo samples used in previous studies [23, 27]. The simplified steps for HTL induction of chicken embryos are as follows: Translate the fertile white leghorn chicken eggs (obtained from the China Agricultural University) [28] into a 37 ℃ humidified incubator and incubate for 28–30 h (i.e., E1-E2 stage). Then, inject 0.5 µl of 0.5 mM HTL solution into the neural groove under a dissection microscope and seal the egg tightly. Continue to incubate until the E2, E4, E5, or E8 stages and observe the chick embryo phenotype. The 0.5 mM HTL solution was obtained by diluting HTL (H6503, Sigma) in Tyrode’s buffer (T1420, Solarbio, China), which includes 134 mM NaCl, 0.34 mM Na2HPO4, 2.9 mM KCl, 12 mM NaHCO3, and 20 mM Hepes, and phenol red was added to facilitate observation of the embryo’s condition [29]. The control group was injected with an equivalent volume of Tyrode’s buffer and phenol red.

### NE4C and SV129 Cell Culture and HTL Treatment

E9 mouse neural stem cells (NE4C) (SCRC-CRL-2925™) were cultured in pre-coated poly L-lysine T25 plates with passaging medium (90% MEM (11,090,073, Thermofisher); 10% FBS (10099141C, Thermofisher)) [23]. E4.5 SV129 cells were provided by Xuanwu Hospital, pre-coat gelatin plates, growth medium: 85% DMEM, etc. [29]. Cells are cultured in a 37 °C humidified environment with 5% CO2, and the passage ratio is 1:4 to 1:7. After starvation treatment (using 1% FBS medium for 24 h), cells are incubated in FBS medium containing 0.5/1 mM HTL for 8 h, while untreated cells serve as controls.

### *Men1/Mars* Overexpression in NE4C

Mouse *Men1* cDNA (NM_001168488) (containing 1854 bp CDS) was cloned into a pcDNA3.1 vector (purchased from Sangon Biotech, China); And mouse *Mars* cDNA (NM_004990) was cloned into a pRK7 vector, with 2700 bp ORF size (Sangon Biotech) [13]. The cells were initially cultured until they reached 30–40% confluence in a medium free from antibiotics before undergoing transfection with *Men1*- and *Mars*-specific plasmids by Lipofectamine transfection reagent (L3000075, Thermofisher), all carried out following the guidelines provided by the manufacturer. Concurrently, an empty vector was employed as a control measure. Post incubation for a period spanning 24 to 96 h, the cells were collected for use in subsequent experiments. The grouping information is as follows: cells transfected empty vector as “NC” group, cells transfected *Men1* cDNA as “*Men1* ( +)” group, cells transfected *Mars* cDNA as “*Mars* ( +)” group.

### Western Blotting (WB)

Histones were extracted by acid extraction method from the tissues or cells [23, 30]. Total proteins were extracted by using the Protein Extraction Kit (C006225, Sango Biotech). Ten-microgram protein samples per lane were separated by 4–12% NuPAGE™ Bis–Tris gel (NP032B, Thermo Scientific™). Primary antibodies used were as follows: DNA Damage Antibody Sampler Kit (#9947, Cell Signaling Technology (CST)), including phospho-ATR (Ser428) (#2853, CST), phospho-ATM (Ser1981) (#5883, CST), phospho-Chk2 (Thr68) (#2197, CST), phospho-Chk1 (Ser345) (#2348, CST), phospho-Histone H2A.X (Ser139) (#9718, CST), ATR (#13,934, CST), and CHK1 (#2360, CST), and SET1/COMPASS Antibody Sampler Kit (#25,501, CST), including Mll1 (#14,689, CST), Menin (#6891, CST), and Wdr82 (#99,715, CST); MARS (ab180497) was purchased from Abcam. In addition, we also used H3K4me3 (#9751, CST), H3K79hcy [23], H3K79me2 (#5427, CST), H2AX (#7631, CST), and H3 (Ab267372, Abcam) for detection of histone modifications. The intensity of blots was quantified with densitometry using by using an Image Lab software (Gel Image System, Tanon, Shaihai, China).

### Immunofluorescence (IF)

Cells were firstly washed with 1 × PBS twice and treated as follows: fixation with 0.5% paraformaldehyde for 15 min and then permeabilized in 0.5% Triton X-100 for 20 min at room temperature and blocked with 1 × PBS containing 10% normal goat serum and 0.3 M glycine for 60 min. Cells were incubated with γ-H2A.X antibody diluted in 5% normal goat serum overnight at 4 °C. After washing, secondary antibodies with Alexa Fluor 488 (ab150077, Abcam) were used for 1-h incubation at room temperature in the dark. Nuclei were counterstained with DAPI. Images were captured on a Zeiss LSM710 confocal microscope.

### ChIP-qPCR

According to the manufacturer’s protocol, SimpleChIP® Enzymatic Chromatin IP Kit (#9002, CST) was used for the ChIP assays. Formaldehyde cross-linked chromatin was obtained from about 1 × 10^7^ cells and further immunoprecipitated with H3K79Hcy/H3K4me3 antibodies overnight at 4 °C. Normal rabbit IgG was used as a negative control. DNA–protein complexes were analyzed by qPCR and primers were designed by Primer 3 in gene promoter regions (Primers used in this experiment are listed in Supplementary Table 2). Relative enrichment of H3K79hcy/H3K4me3 on genes was determined using the 2^(C[T] input sample ‑C[T] IP sample)^_Case_/2^(C[T] input sample ‑C[T] IP sample)^_Control_ method, C[T] = Ct = threshold cycle of the PCR reaction [29].

### Transcription Analysis by RT-qPCR

The TRIzol reagents (15,596,026, Invitrogen) were used for extraction of total RNA from fresh-frozen tissues or fresh-harvested cells. Then, RNA was reverse transcribed using a First Strand cDNA Synthesis kit (K1612, TransGen Biotech). The qPCRs were performed on an Applied Biosystems 7500 Real-Time PCR system (Thermo Fisher Scientific, Inc.) using PCR UltraSYBR Mixture kit (CW0956, CWBIO). The expression levels of the target genes were normalized to *Gapdh* expression levels, and the data was calculated according to the cycle threshold (2^−△△Ct^) method. The primers are listed in Supplementary Table 3.

### Nanostring for mRNA Detection

Total RNA was extracted from E5 chicken embryo brain tissue sample using the RNeasy Plus Kit (Qiagen, Canada). The NanoString nCounter System detection method (NanoString Technologies) was used to examine the transcription levels of genes. Hybridization was performed according to the NanoString Gene Expression Assay manual [31]. Briefly, each target mRNA was made into a unique multiplex probe using two sequence-specific probes. The two probes complementarily construct in a target region of 100 bases. The capture probe consists of a target-specific oligonucleotide and biotin. The reporting probe was composed of the second target-specific oligonucleotide, connected to a unique dye-labeled RNA fragment chain for system detection. Each RNA sample of about 100 ng was mixed with 20 µL of nCounter reporter probes and 5 µL of nCounter capture probes in the hybridization buffer. Purified target/probe complexes were further eluted and immobilized on a cartridge for data collection, which is carried out in the nCounter digital analyzer. Results were normalized against GAPDH, CLTC, and GUSB genes.

### Statistical Analysis

The SPSS software version 25.0 (SPSS Co., Chicago, IL) was used for statistical analysis. The Shapiro–Wilk test was used to assess the normality of all continuous variables. For the normality data (PCR and Western blot data in this study), data were expressed as means ± SEM, and unpaired Student’s two-tailed *t* test was used when comparing two groups; Comparisons among multiple groups were examined by one-way ANOVA plus post-hoc test. For the nonnormal distribution data, they were expressed as medians (interquartile ranges) and were analyzed via the Wilcoxon Mann–Whitney test (two groups) and the Kruskal–Wallis non-parametric test with post-hoc Bonferroni correction for multiple comparisons. For categorical variables, *χ*2 test or Fisher exact probability test was used for the comparisons between groups. Histograms were mainly drew by GraphPad Prism 8.0 (GraphPad Software, USA) and differences were considered statistically significant if the *P* value was less than 0.05.

## Results

### High HTL Induces DNA Damage Accumulation and Inactivates the Atr-Chk1-NER Signaling Pathway

**Fig. 1:**
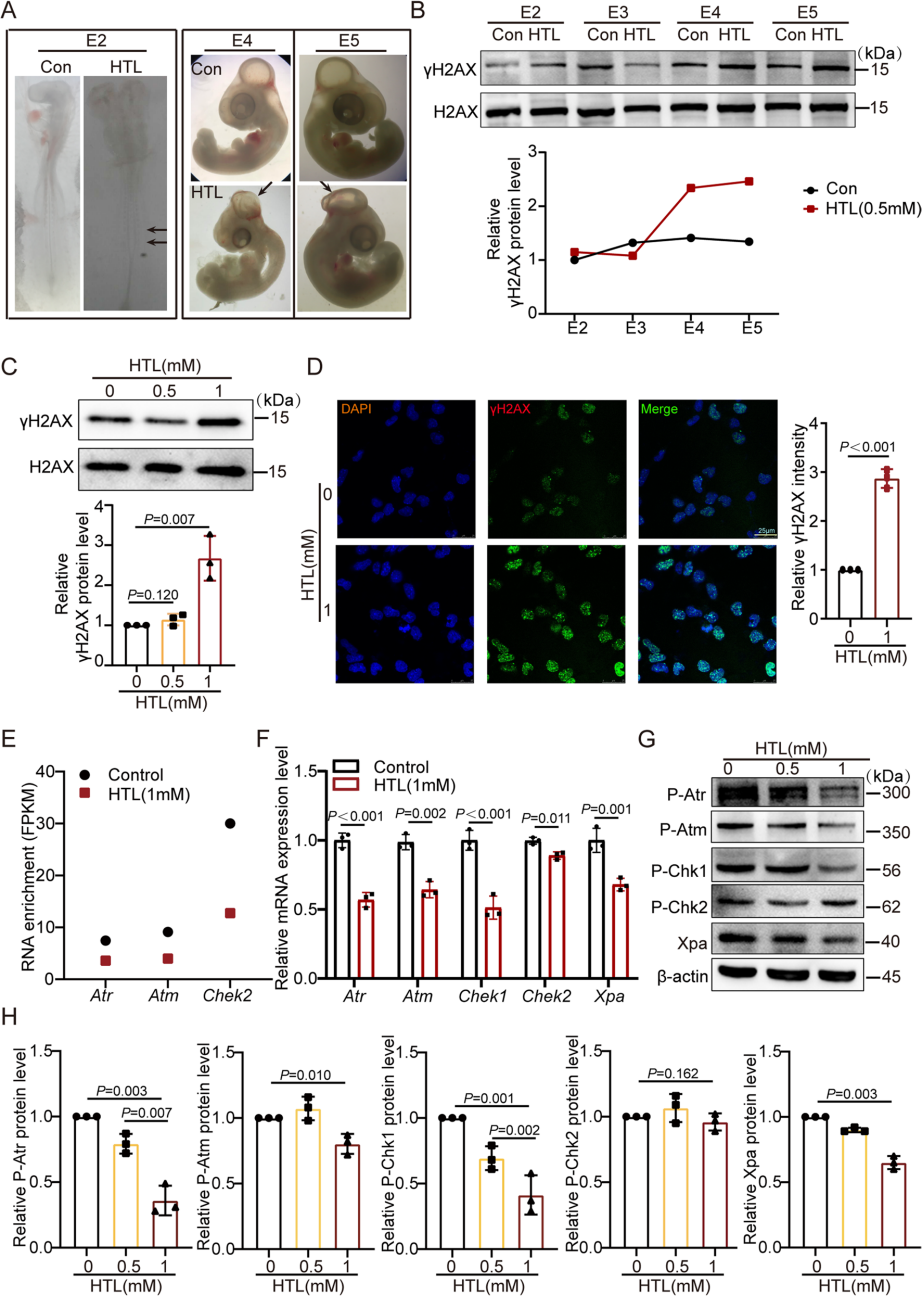
Increase of DNA damage in HTL-treated chicken embryos and NE4C. **A** Eggs treated with 0.5 mM-HTL exhibited spinal bifida aperta at the stage of embryo 2 (E2) (arrow indicated) and neural tube deformities at the stage of E4 and E5. **B** Increase in γH2A.X expression during brain development in high-HTL-treated chicken (*n* = 3). E1, 24 h after embryo incubation; E2, 48 h after embryo incubation; E3, 72 h after embryo incubation; E4, 96 h after embryo incubation; E5, 120 h after embryo incubation. The *P* values were calculated with unpaired *t* test. **C** The expression of γH2A.X in NE4C cells treated with 1 mM HTL was significantly increased compared to those treated with 0 mM or 0.5 mM HTL (*n* = 3). The *P* values were calculated with one-way ANOVA plus post-hoc test. **D** Left panel: IF method confirmed enhanced γH2A.X in 1 mM HTL treated NE4C cells; right panel: quantification of the IF signal intensity shown in scatter plot (*n* = 3). The *P* values were calculated with unpaired* t* test. **E** Decreased RNA content of *Atr*, *Atm*, and *Chek2* in HTL-treated NE4C cells by RNA-seq analysis. **F** RT-qPCR validated decreased mRNA expression level of *Atr*, *Atm*, *Chek1*, and *Xpa* in 1 mM HTL-treated NE4C cells (*n* = 3). The *P* values were calculated with unpaired *t* test. **G**–**H** Reduced protein expression of p-Atr, p-Chk1, and Xpa in 1 mM HTL-treated NE4C cells compared to 0/0.5 mM HTL-treated cells by WB analysis (*n* = 3). The *P* values were calculated with one-way ANOVA plus post-hoc test

We previously established a chicken embryo NTD model via HTL induction; specifically, we studied 107 chicken embryos treated with HTL and found that at E2 (48 h after embryo incubation), no stillbirths were observed, and the rate of embryonic malformations was 59.1%; at E3 (72 h after embryo incubation), the stillbirth rate was 9.09%, and the rate of embryonic malformations was 50%; at E5 (120 h after embryos incubation), the stillbirth rate was 15.1%, and the rate of embryonic malformations was 56.6%; at E8, the stillbirth rate was 10%, and the rate of embryonic malformations was 50%. These embryonic deformities included NTDs, heart defects, cerebral atrophy, and tail deformities. Particularly, the incidence of NTDs was about 36%, of which spina bifida accounted for about 10%, while brain herniation and other brain malformations constituted about 26% [23, 27]. Figure 1A shows typical spinal cord closure deformities at stage E2 and brain developmental malformations at stages E4 and E5. We observed an increase in γH2AX levels in the brain tissues of NTD embryos at stages E4 and E5 induced by high HTL (belong to the 36% of embryos with NTDs) (Fig. 1B), suggesting the occurrence of DNA damage in high HTL-induced NTDs. Moreover, our findings indicated a gradual increase in DNA damage from E3 to E5, implying that DNA damage may have a role in the progression of NTDs. Additionally, we also observed a decrease in the mRNA expression levels of key genes in the DDR pathway, such as *Atr*, *Chek1*, *Xpa*, *Ercc8*, and *Cul4a*, in the brain tissues of E5 chicken embryos after HTL treatment, while the expression levels of *Atm*, *Chek2*, *Ddb1*, *Usp7*, and *Lig1* remained unchanged (Supplementary Fig. 1A). These results indicate that high HTL may lead to DNA damage in vivo and disrupt the pathway of DDR.

We further assessed DNA damage in NE4C cells using WB and IF. An increase in DNA damage was observed in cells treated with 1 mM HTL (Fig. 1C–D). DDR pathway-related genes (*Atr*, *Atm*, *Chek2*) were suppressed, as confirmed by RNA-seq [23] and RT-qPCR (Fig. 1E–F). Additionally, phospho-Atr, phospho-Atm, and phospho-Chk1 protein expressions were reduced in the 1 mM HTL-treated group, whereas phospho-Chk2 remained unchanged (Fig. 1G–H). We also found that the initiator of the NER pathway, Xpa, and its downstream genes *Ercc8*, *Ddb1*, *Cul4a*, *Usp7*, and *Lig1* was significantly downregulated in 1 mM HTL-treated NE4C and SV129 cells (Supplementary Fig. 1B). These findings indicate that high HTL-induced DNA damage in neural stem cells inhibited the DNA damage response Atr-Chk1 pathway (rather than the Atm-Chk2 pathway), leading to an impaired Xpa-driven NER pathway.

### Downregulated Menin-H3K4me3 is an Effector in the High HTL-Induced DNA Damage Response

**Fig. 2:**
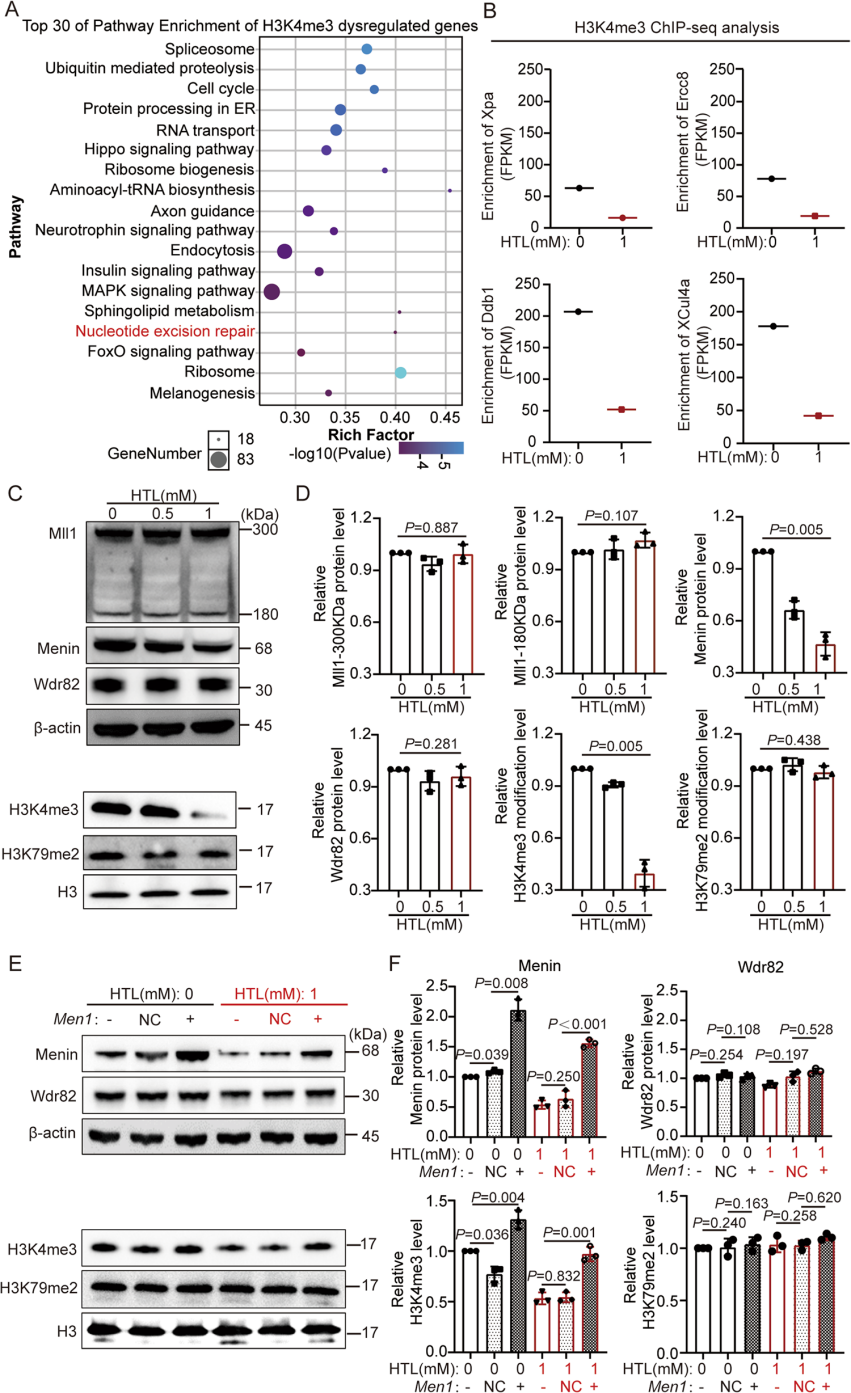
Decreased of Menin regulates declined H3K4me3 level in HTL-treated NE4C cells. **A** The top 30 enriched pathways of H3K4me3 dysregulated genes were analyzed by KEGG. The term “rich factor” refers to the ratio of the number of differentially expressed genes located in a particular pathway entry to the total number of annotated genes located in that pathway entry. **B** H3K4me3 ChIP-seq analysis on NER genes: *Xpa*, *Ercc8*, *Ddb1*, and *Cul4a* in 0 and 1 mM HTL-treated NE4C cells. **C**–**D** WB analysis demonstrated decreased Menin protein expression and H3K4me3 modification in 1 mM HTL-treated NE4C cells (*n* = 3). The *P* values were calculated with one-way ANOVA plus post-hoc test. **E**–**F** Overexpressed of *Men1* in 0 mM and 1 mM HTL-cultured NE4C, respectively, which further induced an increased of H3K4me3 (*n* = 3). The symbol “-” indicates that the cell was not transfected with any plasmid; “NC” denotes that the cell was transfected with an empty plasmid; “ + ” denotes that the cell was transfected with a plasmid overexpressing the *Men1* gene. The *P* values were calculated with one-way ANOVA plus post-hoc test

To investigate the molecular mechanisms of high HTL involved in inhibiting DDR pathways, we reviewed our previous research and found that histone methylation levels change under high HTL conditions. Furthermore, 18 out of 45 genes, including *Ercc8*, *Ddb1*, *Clu4a*, and *Usp7*, in the NER pathway were regulated by H3K4me3 (Fig. 2A–B; Supplementary Table 4–5) [23]. Concurrently, other studies reported that the H3K4me3 methyltransferases Menin and MLL1 regulated DNA damage repair [32, 33]. Taken together, we hypothesize that MLL1/Menin-regulated H3K4me3 has a crucial role in the inhibition of DDR by high HTL. Consequently, we examined the modification level of H3K4me3 and the protein expression levels of related methyltransferases.

WB results indicated that Menin protein expression levels were significantly downregulated in 1 mM HTL-treated NE4C cells (also significantly downregulated in chicken embryonic brain tissue induced by HTL at E5 stage), whereas Mll1 and Wdr82 (other H3K4me3 methyltransferases) remained unchanged (Fig. 2C–D; Figure S2A). This accompanied a decrease in H3K4me3 modification levels (H3K79me2, a control, showed no difference). These data suggest that downregulated Menin protein and H3K4me3 modification levels might be effectors of high HTL. However, it will be necessary to confirm whether H3K4me3 is regulated by Menin under high HTL conditions.

Therefore, we overexpressed the *Men1* gene in normally cultured cells and 1 mM HTL-treated cells (Fig. 2E–F). The results showed that Menin overexpression led to an increase in H3K4me3 modification levels (with no impact on H3K79me2), suggesting that Menin regulates H3K4me3 modification levels under normal and high HTL conditions. Menin protein may be a precursor effector molecule through which high HTL epigenetically regulates DDR pathways via H3K4me3.

### A Decrease in Menin-H3K4me3 Regulates the Dysregulation of the Atr-Chk1-NER Pathway in High HTL

**Fig. 3:**
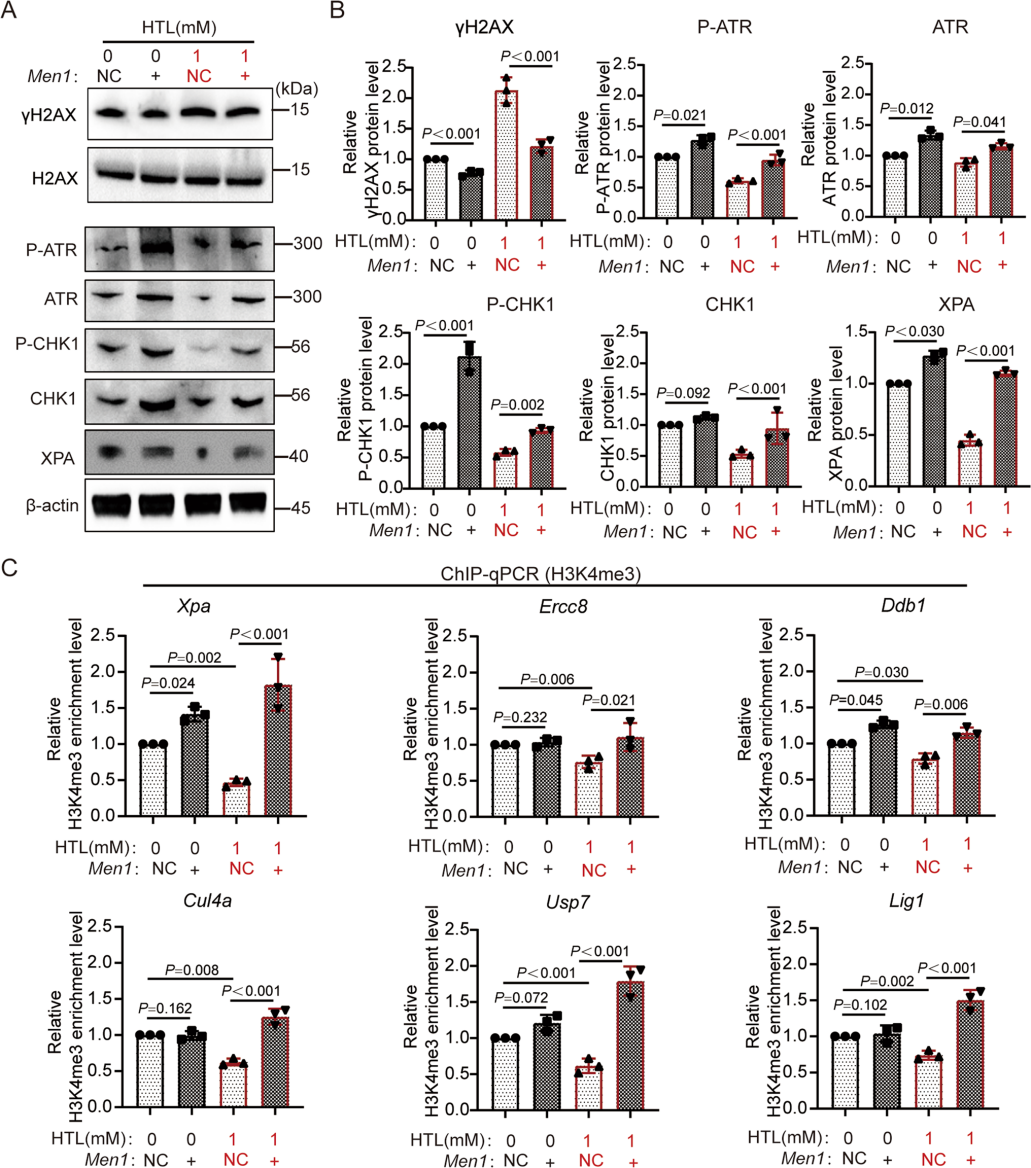
Decreased of Menin-H3K4me3 regulates inhibition of DNA damage response pathway in high HTL. **A**–**B** Decreased γH2A.X and activation of P-Atr-Chk1 in *Men1*-overexpressed cells upon 1 mM HTL treatment (*n* = 3). “NC” denotes that the cell was transfected with an empty plasmid; “ + ” denotes that the cell was transfected with a plasmid overexpressing the *Men1* gene. The *P* values were calculated with one-way ANOVA plus post-hoc test. **C** ChIP-qPCR analysis of histone H3K4me3 enrichment in different regions of NER pathway genes: *Xpa*, *Ercc8*, *Ddb1*, *Cul4a*, *Usp7*, and *Lig1*. Blank plasmid or *Men1* gene overexpression plasmid was transfected into NE4C cells treated with 0 mM HTL and 1 mM HTL, respectively (*n* = 3). The *P* values were calculated with one-way ANOVA plus post-hoc test

To determine the impact of the downregulation of the Menin-H3K4me3 cascade on DDR pathway activity within an HHcy environment, we overexpressed *Men1/*Menin in normal and 1 mM HTL-treated cell groups. Our findings revealed that within the HTL group, Menin overexpression significantly diminished γH2AX levels, thereby reducing DNA damage. Moreover, the expression level and activity of ATR increased with the elevation of Menin protein expression, even under an HHcy environment. P-CHK1 and XPA exhibited similar expression trends, suggesting the impaired DDR pathways under high HTL conditions might be ameliorated by Menin (Fig. 3A–B). Subsequently, we investigated whether the influence of Menin on the DDR pathway was mediated by H3K4me3 epigenetic regulation. Utilizing ChIP-qPCR technology, we observed that, in comparison to the “HTL (0 mM) + *Men1*-NC” group, the binding levels of H3K4me3 at the promoter regions of genes including *Xpa*, *Ercc8*, *Ddb1*, *Cul4a*, *Usp7*, and *Lig1* were markedly downregulated in the “HTL (1 mM) + *Men1*-NC” group. Nevertheless, in the case of concurrent overexpression of *Men1* in cells treated with 0 mM or 1 mM HTL, as compared to their corresponding NC groups, there was a noticeable increase in varying degrees for the binding levels of these genes with H3K4me3 (Fig. 3C). These results suggest that the downregulated Menin-H3K4me3 cascade in a high HTL context disrupted the DDR by epigenetically regulating the expressions of DDR pathway-related genes. Thus, overexpressing Menin might alleviate high HTL-induced cellular damage.

### FA Upregulates the Menin-H3K4me3-DDR Cascade Response

**Fig. 4:**
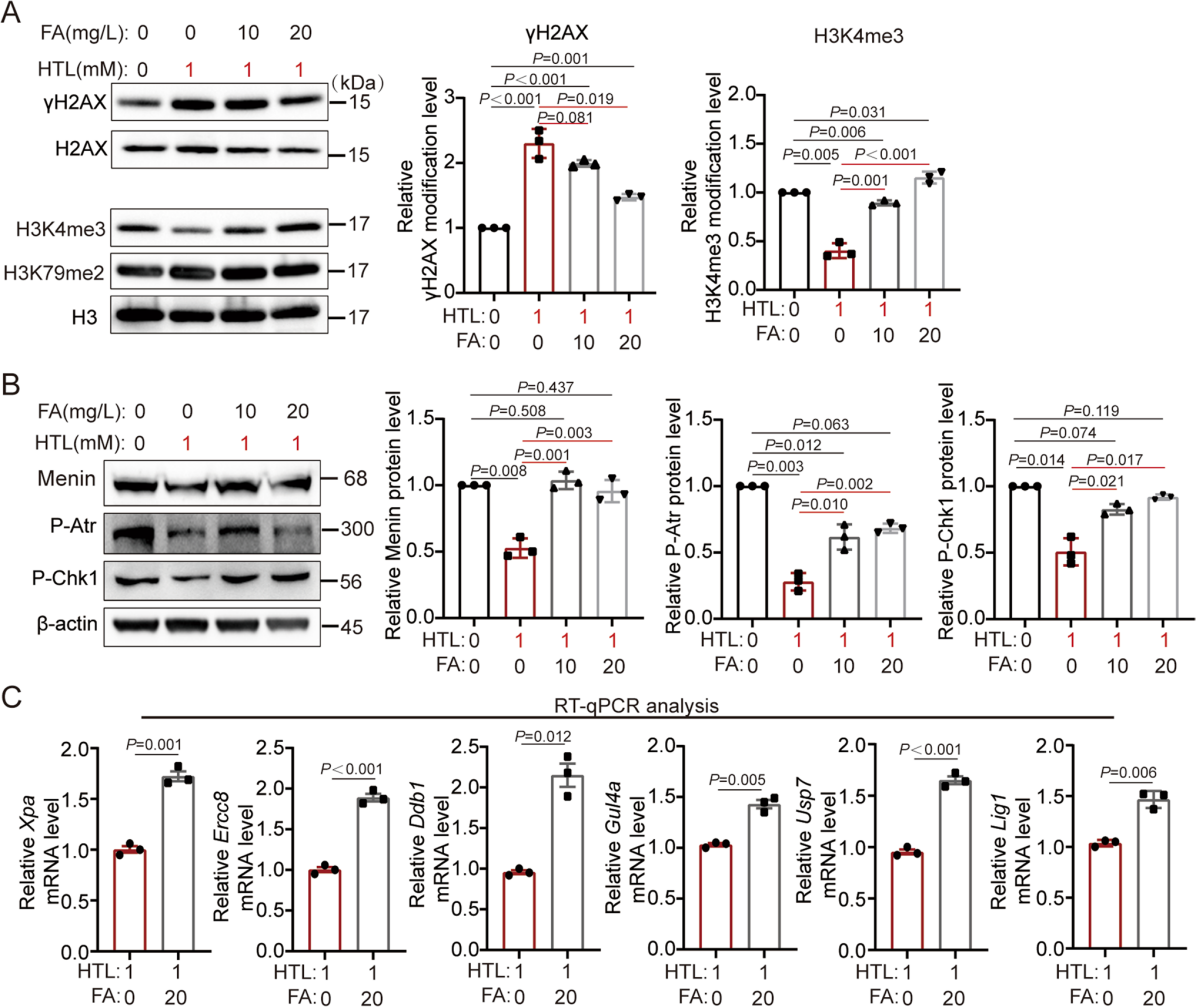
Folic acid (FA) activates ph-ATR-Chk1-NER pathway through increasing expression of Menin. **A**–**B** WB analysis revealed that supplemented with 10/20 mg/L FA in 1 mM HTL-treated NE4C cells can effectively attenuate DNA damage, elevating Menin protein expression and H3K4me3 modification level and enhancing P-Atr-Chk1 activation (*n* = 3). The *P* values were calculated with one-way ANOVA plus post-hoc test. **C** The down-regulation of NER pathway genes RNA expression caused by high HTL can be counteracted by supplementing with 20 mg/L FA (*n* = 3). The *P* values were calculated with unpaired *t* test

Given that supplementary FA can prevent NTDs [1, 2], we investigated whether FA could alleviate DDR damage induced by HTL, which is distinct from the rescue effect achieved through overexpressing the gene *Men1*. The results of WB and IF assays demonstrated that when cells undergoing 1 mM HTL treatment were concurrently supplemented with 20 mg/L FA, the adverse effects of HTL (including increased levels of the DNA damage indicator γH2AX, decreased Menin, ph-ATR, and ph-CHK1 protein expression levels, and H3K4me3 modification levels) were significantly reversed. It should also be pointed out that the protein expression levels of Menin, P-Atr, and P-Chk1 have essentially returned to levels comparable to those of the control group (HTL0 + FA0) **(**Fig. 4A–B; Supplementary Fig. 2B). Moreover, FA also elevated the expressions of genes in the NER pathway (Fig. 4C). These findings collectively suggest that the Menin-H3K4me3-Atr-Chk1-NER pathway might be a therapeutic target for FA supplementation to mitigate the adverse outcomes of high HTL.

### Elevated H3K79hcy Might Regulate the Decreased Expression of Menin in High HTL

**Fig. 5:**
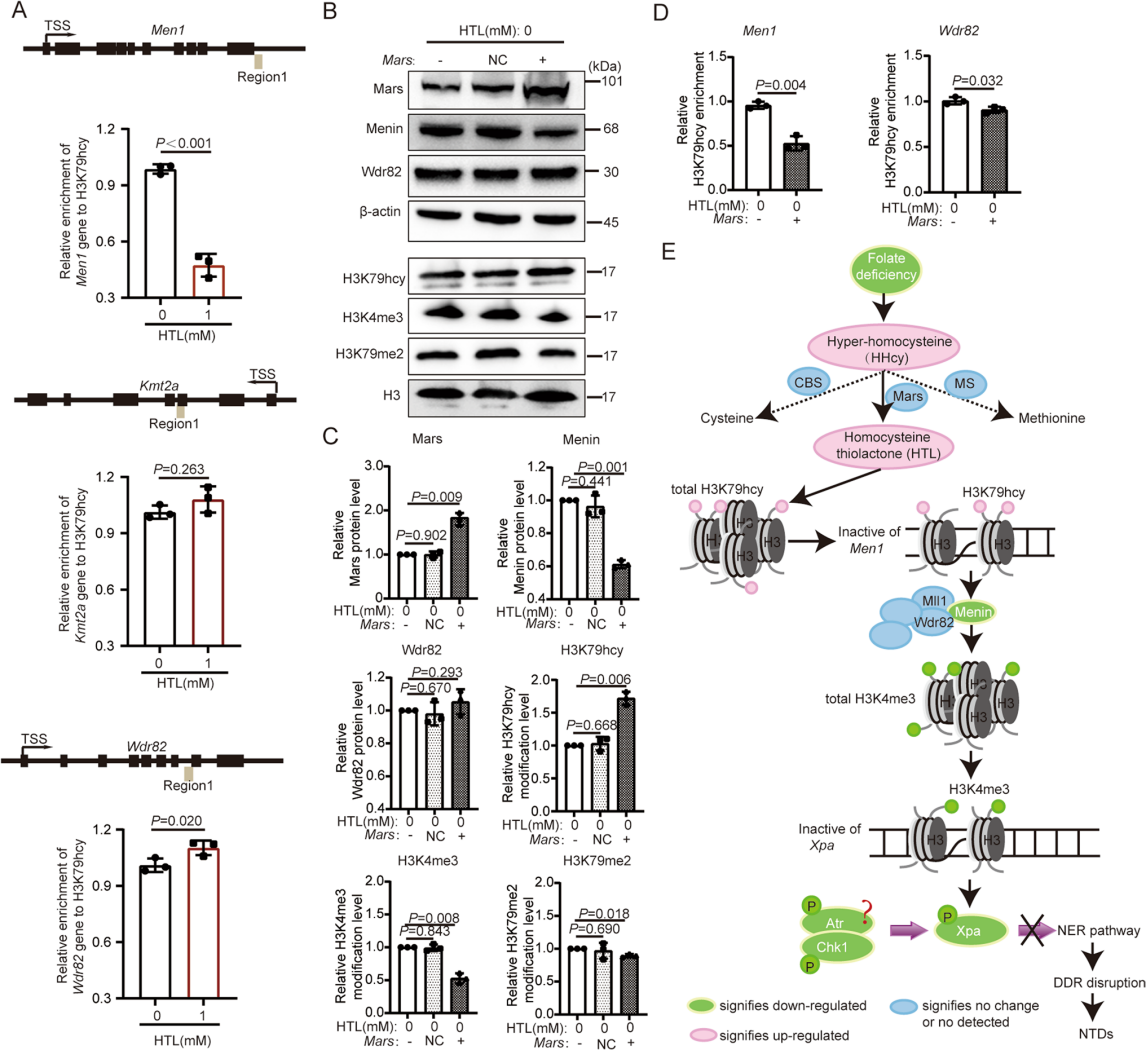
Elevation of H3K79hcy may cause inhibition of Menin in high HTL. **A** The binding levels of H3K79hcy with Men1, Kmt2a, and Wdr82 genes in different regions were analyzed using ChIP-qPCR (*n* = 3). The *P* values were calculated with unpaired *t* test. **B**–**C** Overexpressed *Mars* in normal cultured NE4C induced elevation of H3K79hcy and reduction of Menin and H3K4me3 (*n* = 3). The symbol “-” indicates that the cell was not transfected with any plasmid; “NC” denotes that the cell was transfected with an empty plasmid; “ + ” denotes that the cell was transfected with a plasmid overexpressing the *Mars* gene. The *P* values were calculated with one-way ANOVA plus post-hoc test. **D** ChIP-qPCR assays validated reduced enrichment of *Men1* with H3K79hcy in *Mars* overexpressed cells (*n* = 3). The *P* values were calculated with unpaired *t* test. **E** Pathogenic mechanism diagram of HHcy or high HTL leading to NTDs: The formation of HHcy or high HTL could be due to deficiency of folate. This condition increases the level of H3K79hcy modifications, thus epigenetically regulating the expression of the *Men1* gene, leading to a reduction in Menin protein expression. With the downregulation of Menin, the modification level of H3K4me3 also decreases correspondingly. Such changes further suppress the DDR pathway of P-ATR-CHK1-NER and may potentially participate in the molecular mechanism of NTDs. CBS, Cystathionine Beta-Synthase; H3, Histone 3; P, phosphorylation

Considering the essential role of Menin in the high HTL-induced impairment of the DDR pathway, we investigated how high HTL leads to the downregulation of Menin. High HTL elevated the levels of H3K79hcy modifications in human and chicken embryo models of NTD and epigenetically regulated changes in the expressions of several critical NTD genes [23]. Moreover, Menin is a crucial NTD gene [34], suggesting it might also be regulated by H3K79hcy under high HTL conditions. We retrospectively analyzed published H3K79hcy-ChIP-seq data and found that the three previously validated H3K4me3 modifying transferase genes: *Men1* (encoding Menin protein), *Wdr82*, and *Kmt2a* (encoding Mll1 protein) were enriched by H3K79hcy (Supplementary Table 6). Through ChIP-qPCR analysis, we confirmed that the binding level of H3K79hcy to *Men1* is significantly reduced in cells treated with HTL compared to control cells, while the binding level with *Kmt2a* does not show a significant change. However, its binding level with *Wdr82* has slightly increased (Fig. 5A). This suggested that elevated H3K79hcy might suppress *Men1* expression through an epigenetic modification mechanism.

Next, we overexpressed the *Mars* gene, which encodes a key enzyme responsible for converting the Hcy to HTL (Fig. 5E) [35], in normally cultured NE4C cells. We observed increased H3K79hcy modification levels and decreased Menin protein expression, accompanied by a reduction in H3K4me3 modification levels (Fig. 5B–C). We used ChIP-qPCR to confirm the enrichment of H3K79hcy to *Men1* and *Wdr82* genes was significantly reduced compared with the control group after *Mars* was overexpressed (Fig. 5D). Additionally, we confirmed that overexpression of *Mars* led to the accumulation of DNA damage (Supplementary Fig. 2). In summary, our results suggest that H3K79hcy epigenetic modifications may be a regulatory mechanism for the decreased expression of Menin in high HTL (Fig. 5E).

### Menin-H3K4me3 Levels Are Decreased in HHcy-Related Human Fetal NTD Samples

**Fig. 6:**
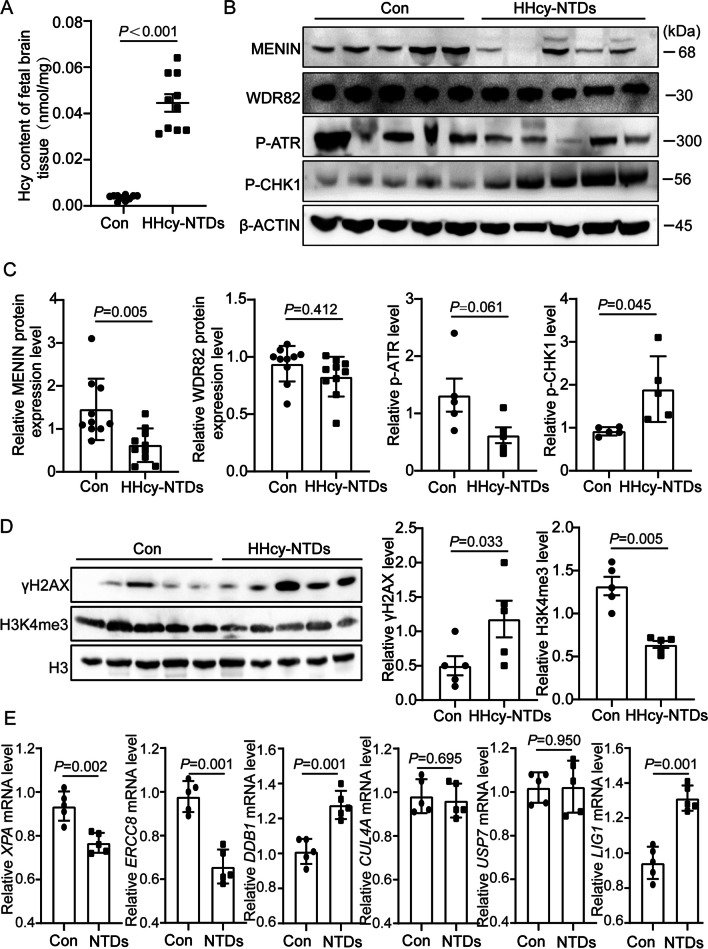
Validation of decreased protein expression of MENIN and P-ATR in HHcy-related human fetal NTDs. **A** Hcy levels in 10 normals and 10 NTDs from human fetal brain tissues. **B**–**C** Decreased of MENIN, whereas increased P-CHK1 protein expression and the decreasing trend of P-ATR in HHcy-related human fetal NTDs (*n* = 10). **D** WB validated an increased of γH2A.X and decreased of H3K4me3 in HHcy related human fetal NTDs (*n* = 5). **E** RT-qPCR assays for detection mRNA level of NER pathway genes: *XPA*, *ERCC8*, *DDB1*, *CUL4A*, *USP7*, and *LIG1* in brain tissues of human fetal NTDs (*n* = 5). All the *P* values were calculated with unpaired *t* test

We selected 10 fetal NTD samples from patients with HHcy (0.0446 ± 0.0038 nmol/mg tissue) in fetal brain tissue and 10 controls with normal Hcy content (0.0036 ± 0.00036) (*P* < 0.001) (Fig. 6A). WB assay demonstrated that in the HHcy-NTD group, there is a significant decrease in the expression level of Menin protein. Although the expression of phosphorylated ATR exhibited a downward trend, the difference was not statistically significant. However, the phosphorylation of CHK1 has notably increased. Additionally, γH2AX expression levels were significantly elevated, but H3K4me3 was markedly downregulated (Fig. 6B–D). Among the six NER genes, mRNA levels of *XPA* and *ERCC8* were decreased; however, levels of *DDB1* and *LIG1* were increased, and no significant changes were observed for *CUL4A* and *USP7* (Fig. 6E). These results partially describe the mechanism of ATR inactivation in HHcy-induced NTDs.

## Discussion

Elevated maternal homocysteine levels (hyperhomocysteinemia, HHcy) contribute to an increased incidence of fetal NTDs [1]. Our study demonstrated that the expression of Menin was markedly downregulated in high HTL-induced chicken embryos and HHcy-related human NTD brain tissue samples. This leads to the further disruption of the ATR-Chk1 signaling pathway and Xpa-mediated NER, a crucial DDR mechanism, via H3K4me3-mediated epigenetic regulation. Moreover, Menin itself may be subjected to epigenetic modulation by H3K79hcy (Fig. 5E). Collectively, these findings shed light on the underlying molecular mechanisms through which HHcy/high HTL promotes the development of NTDs.

In this study, we initially observed an escalation in DNA damage in E4 and E5 chicken embryos, induced by high HTL treatment; moreover, the expression levels of DNA damage repair pathway genes such as *Atr*, *Chek1*, *Xpa*, and *Ercc8* were significantly reduced in chicken embryos at the E5 stage; subsequently, γH2AX was augmented in NE4C cells with high HTL, and the DDR pathway (Atr-Chk1-NER) was compromised. Additionally, we discovered a decline in P-ATR and downregulation of *XPA* and *ERCC8* gene expressions in human fetal NTDs associated with HHcy. Collectively, high HTL/HHcy resulted in an increase in DNA damage in vitro and in vivo, as well as disruption of the P-ATR-related NER pathway (Fig. 1; Supplementary Figs. 1 and 6). However, in human fetal NTDs associated with HHcy, the expression of P-CHK1 protein was enhanced and *DDB1* and *LIG1* mRNA were upregulated, results that are contrary to those obtained for in vitro NE4C with high HTL; Moreover, this difference can also be seen in the E5 stage chicken embryos induced by high HTL, reflected in that the mRNA expression levels of pathway genes *Ddb1*, *Usp7*, and *Lig1* do not change (Supplementary Fig. 1B). We hypothesize that the observed diversity in the expression of DDR pathway genes under high HTL/HHcy conditions across various species is due to two reasons. First, although high HTL is a significant route for intrabody HHcy, other paths exist for HHcy, such as its conversion into cysteine by cystathionine β-synthase or its regeneration into methionine via methionine synthase (Fig. 5E). This suggests that a high HTL environment cannot fully simulate a HHcy environment. Second, the inherent heterogeneity and differences in developmental stages within organisms—for instance, inconsistent time points for gene testing—might contribute to the variations in experimental results. In this study, conducting such tests in vitro in mouse NE4C at the E9 stage (i.e., when the mouse is in the neural tube closure period [36]) is vastly different from tests conducted in chicken embryos at the post-incubation E5 stage (the neural tube closure period for chicken embryos is begin within 33 to 36 h after incubation and complete within 48 to 72 h [37]). Not to mention, the NTD fetal samples we collected at approximately 20 weeks of gestation, which surpasses the stage of neural tube closure (usually commencing 25–27 days after fertilization and completed between 28–30 days [36]). Therefore, the difference in DDR gene expression may be due to chicken embryos and human samples being at significantly mature developmental stages. Despite these differences, the increase in DNA damage caused by high HTL/HHcy and the dysregulation of the DDR pathway are widely observed pathological phenomena both in vivo and in vitro.

Subsequently, we found that Menin expression was inhibited under high HTL/HHcy conditions and H3K4me3 modification levels were diminished, exerting a pivotal regulatory function related to the disturbance of the Atr-Chk1-NER pathway (Figs. 2, 3 and 6). Moreover, we attempted to mitigate the adverse impacts of high HTL by gene therapy and nutritional supplementation approaches; the results demonstrated that Menin overexpression or FA supplementation enhanced DDR via H3K4me3 epigenetic regulatory mechanisms (Figs. 3 and 4). Finally, we investigated the mechanisms related to the reduction of Menin under high HTL conditions and postulated that H3K79hcy [23] might have a negative effect on the epigenetic regulation of Men1/Menin expression (Fig. 5). Nevertheless, further exploration is required to ascertain whether other mechanisms modulate the aberrant expression of Men1/Menin and if additional histone modification mechanisms participate in the disruption of DNA damage repair responses in high HTL/HHcy.

The protein Menin, encoded by the *Men1* gene, is predominantly a nuclear scaffold protein that has a crucial role in embryonic development. When the *Men1* gene is a null mutant in mice, a significant proportion of embryos exhibit neural tube exposure, leading to NTDs [34]. The pathological mechanism of menin-induced NTDs remains unclear, but currently, our understanding of the functional mechanisms of Menin can be summarized as follows: (1) Menin interacts with various proteins to regulate gene transcription levels: it associates with transcription activators such as c-myb and H3K4me3 methyltransferases such as Mll2; with transcription repressors such as deacetylase Sirt1 and H3K27me3 modifying enzyme EZH2; and with key molecules of critical signaling pathways involved in neural tube closure, such as β-catenin in the Wnt pathway [38] and PRMT5 protein in the SHH pathway [39], thereby regulating gene expression or pathway activity [40, 41]. (2) Menin participates in DNA damage repair: in pancreatic neuroendocrine tumors or lung cancer tissues, the low expression of Menin triggered abnormal DNA damage responses, increased γH2AX staining, inhibited the p-ATR pathway, and activated the p-ATM pathway to maintain genomic stability [32]. In our study, we found that in high HTL-induced NTD, low Menin expression further suppressed H3K4me3, which regulated the expressions of genes associated with the Atr-Chk1-NER DNA damage repair pathways. This research further enhances our understanding of the Menin-regulated DDR mechanism and gene expression. However, in high HTL/HHcy-induced NTDs, the expression pattern and regulatory mechanisms of Menin are still unclear.

We previously identified elevated H3K79hcy modification levels in high HTL-induced NTD chicken embryos, which led to the inhibition of NTD-associated gene expression [23]. Building on this work, through the in vitro overexpression of the *Mars* gene to upregulate H3K79hcy modification, we confirmed its crucial epigenetic regulatory role in Menin expression. It is important to consider that an excessive accumulation of AdoHcy (a precursor of Hcy within one-carbon metabolism) can be detrimental because it inhibits most methyltransferases. This indicates that HHcy might also repress Menin expression via this mechanism [42]. Moreover, although Hcy is a non-protein amino acid, HHcy-HTL triggers protein homocysteinylation, a post-translational modification that results in proteins losing their inherent biological function leading to adverse effects on various disease phenotypes [43]. Many novel N-homocysteinylated protein modifications have been reported in NTDs, including N-homocysteinylated SOD1/2 [13]. Although there is no direct evidence for Menin homocysteinylation, Menin can undergo SUMOylation, phosphorylation, and ubiquitylation [40]. Consequently, we propose that the N-homocysteinylation of Menin might represent an alternative regulatory mechanism contributing to the reduction of Menin expression in high HTL-induced NTDs and, thus, warrants further study.

There is interplay between H3K79hcy and H3K4me3 and interactions between histone post-translational modifications have been reported for H3K4me3 and H3K27ac [44], as well as H3K4me3 and H4K16ac [45], indicating crosstalk between epigenetic mechanisms is common. In contrast to H3K4me3, which is primarily located at active promoters, enhancers, or near transcription start sites and is involved in gene activation, H3K79hcy preferably resides within gene bodies and is mainly associated with gene repression [23, 46]. Our findings demonstrated that there is a concomitant upregulation of H3K79hcy and downregulation of H3K4me in HHcy-induced NTDs, suggesting a potential interplay between these two modifications. Furthermore, high HTL treatment or *Mars* overexpression elevated H3K79hcy levels but downregulated *Men1* expression via negative epigenetic regulation. This, in turn, led to a decrease in Menin protein expression and subsequent reduction in H3K4me3 modification. Considering that Menin itself can bind to histone-modifying enzymes and affect other histone modification levels [40, 47], this raises the question of whether menin might reciprocally influence H3K79hcy levels. This possibility warrants further investigation. However, the underlying mechanism by which this crosstalk is facilitated through menin remains to be elucidated.

There are some limitations in this study that need to be noted. Firstly, in this study, we investigated spina bifida malformations in chicken embryos and human embryos. Due to the relative ease of acquiring brain tissue over spinal cord tissue, their brain tissues were used as research subjects. However, some literature suggests that despite both brain and spinal cord tissues being parts of the central nervous system, their developmental mechanisms may be independent and differ from one another [48]. Furthermore, different NTD phenotypes may arise from distinct etiologies [49]. In light of this, more rigorous approaches should be adopted when selecting and validating models in the future. Secondly, the number of human fetal NTD samples related to HHcy that we have collected is relatively small, so it is necessary to further increase the sample size to consolidate our research findings. Thirdly, whether the dysregulated expression of P-ATR-CHK1 under high HTL or HHcy conditions is also subject to regulation by the Menin-H3K4ME3 mechanism or there exist other regulatory mechanisms (Fig. 5E) is worth further exploration.

In summary, the present study reports that the downregulation of Menin during embryonic development epigenetically modulated the P-Atr-Chk1-NER DNA damage repair pathway through H3K4me3, providing novel insights into the pathogenesis of HHcy-induced NTDs. The investigation of Menin protein offers potential therapeutic targets for the prevention and treatment of NTDs. Furthermore, the regulatory role of H3K79hcy on H3K4me3 provides new evidence of crosstalk between histone epigenetic modification mechanisms.

## Supplementary Information

Below is the link to the electronic supplementary material.

Supplementary file1 (JPG 1265 KB)

Supplementary file2 (JPG 1413 KB)

Supplementary file3 (DOCX 15 KB)

Supplementary file4 (DOCX 15 KB)

Supplementary file5 (DOCX 15 KB)

Supplementary file6 (XLSX 490 KB)

Supplementary file7 (XLSX 87 KB)

Supplementary file8 (XLSX 122 KB)

## Data Availability

The data that support the findings of this study are available from the corresponding author upon reasonable request.
