# Supplementary figures and images for: High Homocysteine-Thiolactone Leads to Reduced MENIN Protein Expression and an Impaired DNA Damage Response: Implications for Neural Tube Defects
Source: Mol Neurobiol. 2024 Feb 22;61(10):7369–83. doi: 10.1007/s12035-024-04033-7 (PMC11415403; doi:10.1007/s12035-024-04033-7)

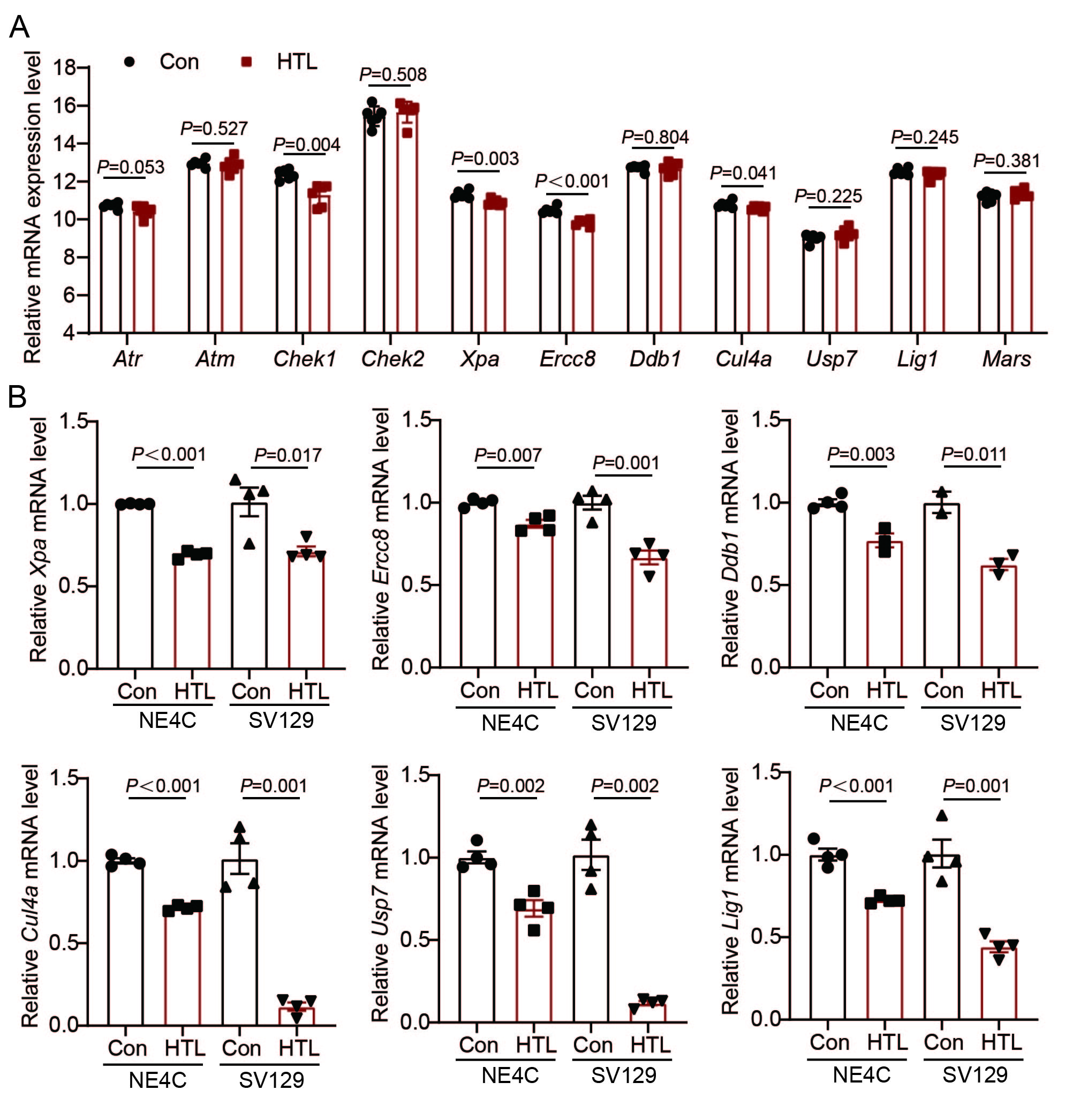

Supplement: Supplementary file 1 — Supplementary file1 (JPG 1265 KB) [file 12035_2024_4033_MOESM1_ESM.jpg]

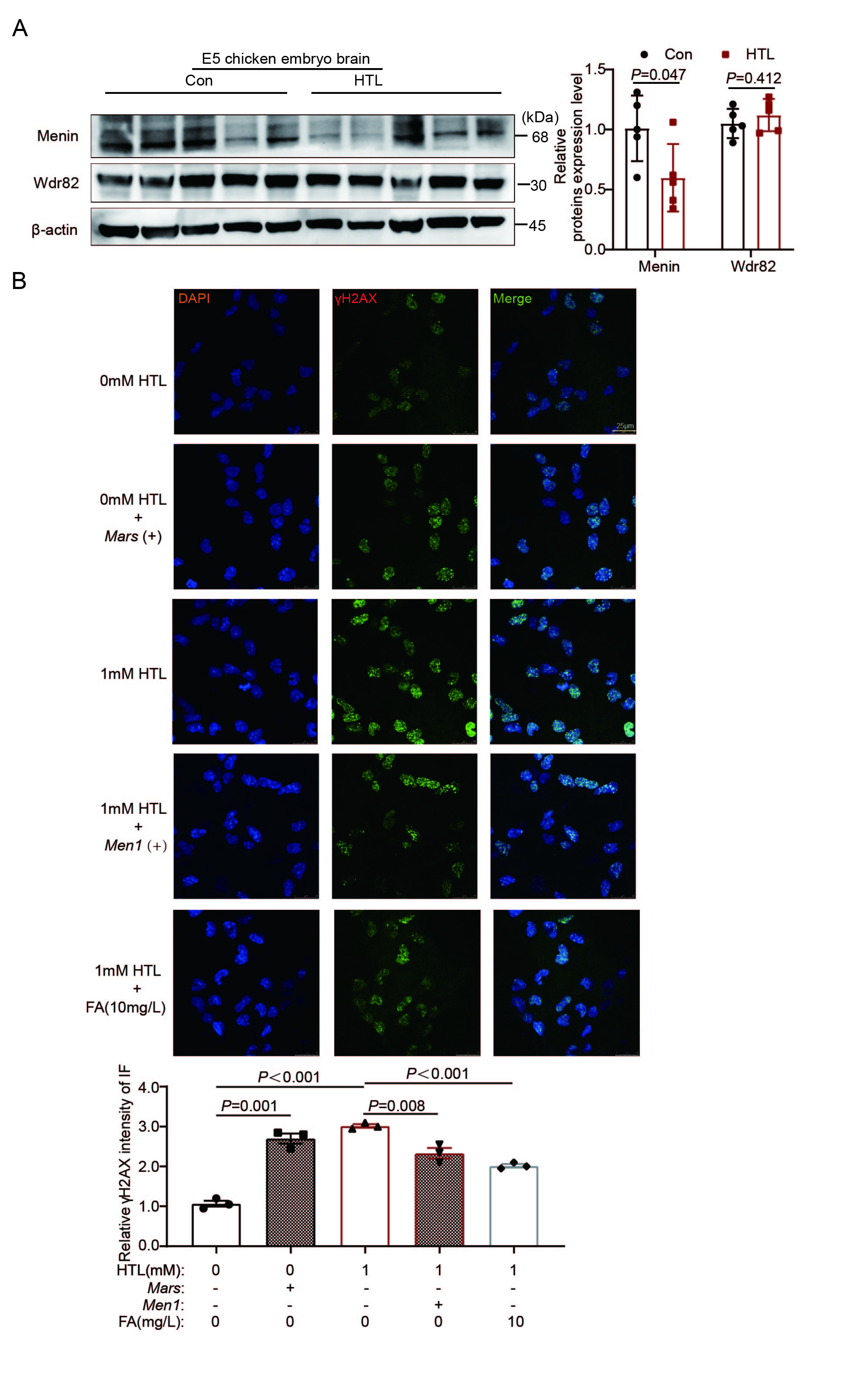

Supplement: Supplementary file 2 — Supplementary file2 (JPG 1413 KB) [file 12035_2024_4033_MOESM2_ESM.jpg]
