# Supplementary material for: High Homocysteine-Thiolactone Leads to Reduced MENIN Protein Expression and an Impaired DNA Damage Response: Implications for Neural Tube Defects
Source: Mol Neurobiol. 2024 Feb 22;61(10):7369–83. doi: 10.1007/s12035-024-04033-7 (PMC11415403; doi:10.1007/s12035-024-04033-7)
Supplement: Supplementary file 3 — Supplementary file3 (DOCX 15 KB) [file 12035_2024_4033_MOESM3_ESM.docx]

| Group | Maternal age, year | Gestational week | Fetal gender, no. (%) | | Concentration of hcy in fetal brain tissues, nmol/mg |
| --- | --- | --- | --- | --- | --- |
|  |  |  | Male | Female |  |
| Control (n=10) | 27.20±0.57 | 19.40±0.65 | 4 (40) | 6 (60) | 0.0036±0.00036 |
| HHcy-NTDs (n=10) | 26.60±0.91 | 24.204±1.05 | 5 (50) | 5 (50) | 0.0446±0.0038 |
| *P*-value | 0.584 | 0.001 | 1.000 | | ＜0.001 |

**Supplementary Table 1**. Clinical characteristics in two human groups of the study.
