# Supplementary material for: High Homocysteine-Thiolactone Leads to Reduced MENIN Protein Expression and an Impaired DNA Damage Response: Implications for Neural Tube Defects
Source: Mol Neurobiol. 2024 Feb 22;61(10):7369–83. doi: 10.1007/s12035-024-04033-7 (PMC11415403; doi:10.1007/s12035-024-04033-7)
Supplement: Supplementary file 4 — Supplementary file4 (DOCX 15 KB) [file 12035_2024_4033_MOESM4_ESM.docx]

**Supplementary Table 2**. Primers for ChIP-qPCR

| Genes | Primer (5'→3') | Target region | Product size (bp) |
| --- | --- | --- | --- |
| *Kmt2a* | F:AATGATGAGGGCCTTTGACG  R:ACCACTGCTGTCATACACCA | chr9:44,833,065-44,833,170 | 106 |
| *Men1* | F: CAAGGGCCTCTGAACTCTGA | chr19:6,340,160-6,340,305 | 146 |
|  | R: AAGGCTCCTAGTCACTGCAG |  |  |
| *Wdr82* | F: TGCACATCTACTCACCAGCA | chr9:106,186,087-106,186,255 | 169 |
|  | R: GACAATGAAGGGACACAGGC |  |  |
| *Ercc8* | F: CACTGCCAACATGACTGCTT | chr13:108159199-108159326 | 128 |
|  | R: CACAGTCAAAGCATGCAGGT |  |  |
| *Ddb1* | F: CTCTCCAGACCCACCCTTTT | chr19:10606200-10606308 | 109 |
|  | R: CGCACACAAACAAGGCAAAG |  |  |
| *Cul4a* | F: GAGGTGTACGTTTTCCCAGC | chr8:13106532-13106653 | 122 |
|  | R: CACAGTCAAAGCATGCAGGT |  |  |
| *Usp7* | F: TCCATTCATTGCCCCTCACT | chr16:8737269-  8737392 | 124 |
|  | R: TCTCATGCTGGGTTGGTCAT |  |  |
