# Supplementary material for: High Homocysteine-Thiolactone Leads to Reduced MENIN Protein Expression and an Impaired DNA Damage Response: Implications for Neural Tube Defects
Source: Mol Neurobiol. 2024 Feb 22;61(10):7369–83. doi: 10.1007/s12035-024-04033-7 (PMC11415403; doi:10.1007/s12035-024-04033-7)
Supplement: Supplementary file 5 — Supplementary file5 (DOCX 15 KB) [file 12035_2024_4033_MOESM5_ESM.docx]

**Supplementary Table3**. Primers for RT-qPCR.

| Genes | Primer (5'→3') | Product size (bp) |
| --- | --- | --- |
| *Xpa* | F: AGCTACAGGTGGTAAAGCGG | 150 |
|  | R: CTGCTTCTTATTGCTCGCCG |  |
| *Ercc8* | F: CTCCAGCCAAATCCCAGTTA | 193 |
|  | R: AGCCAGGGTTACATCGTGAG |  |
| *Ddb1* | F: TCGGAGACTGGCATCATTGG | 211 |
|  | R: CCCCTGAGGATCCTGGTAGA |  |
| *Cul4a* | F: ACACACACGAAGCACCTGAG | 246 |
|  | R: GGAACGAGAGCTTGTGAAGG |  |
| *Usp7* | F: AAGGTGTGGTGGCAGTTAGG | 185 |
|  | R: TCCAAGGAATTCCACCTGAG |  |
| *Lig1* | F: GCCCGGACATTTGAGAAGAT | 115 |
|  | R: GAACAGGGAGCAAGTCTGGA |  |
| *Atr* | F: TTTGCCTGATCATCCAGAATTAG | 125 |
|  | R: TTTCGTGTTGAATTGCTTTCATAG |  |
| *Atm* | F: ATGGAAAGCTTGGGTGTGAC | 240 |
|  | R: CTTGCTGCAGTAGCACAAGC |  |
| *Chek1* | F: CAAATTTTCCTCCTGGCGTA | 210 |
|  | R: GGCTGGCCAATAAGTTCAGA |  |
| *Chek2* | F: GGCTGTGTTGTGAGCTTTGA | 223 |
|  | R: CCTGGCTATCCTGGAACTCA |  |
| *Gapdh* | F: CAAGTTCAACGGCACAGTCA | 102 |
|  | R: AACTCACTGTGGACAAGGCT |  |
